# Supplementary material for: Are carbon emissions trading and green financial instruments synergistic? -Comprehensive quantitative research based on content analysis
Source: PLoS One. 2024 Mar 7;19(3):e0298601. doi: 10.1371/journal.pone.0298601 (PMC10919601; doi:10.1371/journal.pone.0298601)
Supplement: S1 Data — (ZIP) [file pone.0298601.s001.zip › Data/地级市Z_F工作报告.zip!/╔╜╢1⁄2/▒⌡╓▌╩╨/└┤╫╘╣1⁄2╓┌║┼í╛┬φ┐╦╩2╛▌═°í┐_2012.docx]

各位代表:

　　我代表市人民政府向大会作工作报告，请予审议，并请政协委员和其他列席人员提出意见。

一、2008年以来的工作回顾

本届政府自2008年1月就职以来，抢抓“黄蓝”两区建设重大机遇，经受住了金融危机冲击、宏观环境趋紧、旱涝自然灾害频发的严峻考验，开拓创新，科学实干，圆满完成了市九届人大以来的各项目标任务，经济社会呈现出一系列新的积极变化，实现了“十二五”的良好开局。

（一）综合实力显著增强

地区生产总值由2007年的1030.3亿元增长到1817.6亿元，按可比价格计算，增长63%，年均增长13%。

地方财政收入由59.37亿元增长到130.76亿元，增长120.2%，年均增长21.8%。地方财政支出由84.9亿元增长到201.2亿元，增长136.8%，年均增长24.1%，首次突破200亿元大关。金融机构本外币存款余额由566亿元增长到1309.7亿元，增长131.4%，年均增长23.3%;贷款余额由564.3亿元增长到1256.5亿元，增长122.7%，年均增长22.2%。进出口总额由33.6亿美元增长到66.9亿美元，增长99.3%，年均增长18.8%。固定资产投资由481.8亿元增长到1010.7亿元，增长109.8%，年均增长20.3%。社会消费品零售总额由246.8亿元增长到505.8亿元，增长104.9%，年均增长19.5%。连续三年在全省科学发展综合考核中位列第4名。

（二）产业结构明显优化

三产比例由2007年的10.6:59.9:29.5调整为9.8:53.5:36.7。

粮食生产实现“九连增”，规模以上农业龙头企业由470家增长到625家、“三品”认证由156个增长到275个。工业加快转型升级，主营业务收入过百亿元的企业由4家增加到10家;高新技术产业产值由304.9亿元增长到901亿元，增长195.5%，年均增长28.3%。服务业增加值由303.7亿元增长到667.2亿元，按可比价格计算，增长84.9%，年均增长16.6%。全面完成节能减排目标任务，海河迎查取得全国“十大”重点流域和海河流域“双第一”，顺利实现贝壳堤岛与湿地国家级自然保护区科学调整。

（三）城乡面貌发生巨变

市城区累计完成城市道路、景区提升、园林绿化、夜景亮化等项目828个，整体拆迁城中村33个，“数字滨州”加快建设，功能更加完善，品位明显提升。县城区建设亮点纷呈、日新月异。小城镇建设日趋完善、各具特色。村居环境更加干净整洁，创建市级生态文明村90个。“林水会战”成效显著，累计完成水利工程2667项、标准化方田50.2万亩，新建农田林网136万亩，一次性蓄水能力达到8.7亿立方米，林木覆盖率达到28.5%。我市顺利通过全国双拥模范城市、国家卫生城市复审，荣获全国科技进步先进市、中国优秀旅游城市、省级文明城市称号。“四环五海、生态滨州”、“粮丰林茂、北国江南”的“名片效应”日益彰显。

（四）基础设施更加完善

累计投入184亿元，新建铁路61公里、一级公路151公里、农村公路5090公里，完成重大交通工程16项。疏港公路、滨德高速主体全线贯通。大济路、庆淄路完成改造提升。滨港铁路一期运营良好、二期成功奠基，德大铁路（滨州段）工程全面铺开。滨州港上升为区域性重要港口，2个3万吨级码头及附属工程达到靠泊条件，初步形成了陆海相连的交通运输体系。

（五）改革开放扎实推进

行政、人事、财税等制度建设向深层次推进，市县政府机构改革、国有企业改制基本完成，事业单位分类改革加快推进。成功举办黄河三角洲高效生态经济区经贸洽谈会、银企合作促进会等一系列重大节会活动。与60多个国家和地区建立经贸合作关系，实际利用外资由2.5亿美元增长到10.4亿美元，增长313%，年均增长42.6%。上市公司由7家增加到14家。黄河三角洲（滨州）国家农业科技园区成功纳入国家“一城两区”发展规划，邹平经济开发区上升为国家级开发区，省级以上开发区成为新的产业和要素聚集区。

（六）社会事业全面进步

城镇居民人均可支配收入、农民人均纯收入由13888元、4986元，分别增加到22540元、8744元，分别增长62.3%、75.4%，年均分别增长12.9%、15.1%。市民活动中心、公共卫生中心、健身中心加快建设。素质教育深入推进，高等教育、职业教育迈出新步伐。人口计生工作连续4年获得省考核一等奖。累计城镇就业19.7万人，农村劳动力转移就业40.6万人次。开工保障性住房2.3万套，完成农房建设、危房改造13万户。顺利完成国家基本药物制度改革，新农合参合率保持在100%，社会保障体系和城乡救助制度逐步完善。提前半年，在全省地级市中率先同步实现新型农村和城镇居民养老保险制度“全覆盖”。加强和创新社会管理，新一届村委会换届选举顺利完成。扎实推进“平安滨州”建设和社会治安综合治理，重视抓好安全生产，认真做好信访工作，不断完善应急管理体系，严厉打击违法犯罪行为，社会保持和谐稳定。对口援建四川禹里任务圆满完成，帮扶重庆奉节工作扎实推进。法制、民宗、广电、外侨、统计、物价、对台、史志、地名、地震、气象、水文、海事、人防、油区管理、国防动员和妇女儿童、老龄、慈善、残疾人等事业健康发展。

(七)行政效能持续提升

自觉接受人大、政协监督，认真听取各民主党派、工商联、无党派人士意见建议，办理人大代表建议335件、政协提案1314件。深化政务公开和监察审计，“市阳光政务服务中心”、公共资源交易中心建成启用，减少行政审批事项47项。坚持依法行政，严格考核奖惩，强化督导检查，激发内力、推动发展的体制机制更加完善。

(八)发展激情空前高涨

连续开展解放思想大讨论和优化发展环境活动，全市上下始终激情满怀，奋发有为，敢于决策，高效工作，取得了令人惊叹的成绩。如今，广大干部群众信心更加坚定，作风更加务实，精神更加振奋，斗志更加昂扬！经过几年的探索与实践，我们不仅积蓄了力量，夯实了基础，而且磨炼了意志，丰富了经验，同时也深深地体会到：必须解放思想、抢抓机遇，才能不辱使命、不负重托；必须勇于创新、敢于突破，才能活力迸发、优势再造；必须不干则已、干则一流，才能提升境界、追求卓越；必须规划先行、环保优先，才能遵循规律、科学发展；必须以人为本、民生至上，才能众志成城、共筑和谐。历程充满艰辛，成就来之不易。滨州之所以能够创造新业绩、实现新发展，是省委、省政府深切关怀的结果，是市委统揽全局的结果，是全市人民开拓奋进的结果。在此，我代表市人民政府，向奉献智慧和力量的全市人民，致以崇高的敬意！向大力支持我们工作的人大代表、政协委员和各民主党派、工商联、各人民团体、无党派人士、驻滨部队、武警官兵、公安民警，国家部委和省直机关、兄弟市和社会各界，以及所有关心、支持、参与滨州改革发展的海内外朋友，表示衷心的感谢!我们也清醒地认识到，工作中还存在一些问题和不足:经济总量较小，结构性矛盾突出，产业布局不尽合理；高层次人才匮乏，创新能力不强；重大基础设施滞后，县域经济不平衡；制约发展的体制机制障碍有待突破；对政府投资项目监管不严、处罚过轻等等。必须采取更加有力的措施，在今后工作中切实加以解决。在这里需要特别说明的是，去年《政府工作报告》提出的工作任务，分解为100大项、187小项，完成率98.4%。城区集中供热、市民文化中心等项目，由于各种原因未能如期全面完成。对此，我谨代表市人民政府，向各位代表并通过你们向全市人民，表示深深的歉意!在今后的工作中，我们将克服困难，奋力推进未完成的任务。

二、今后五年的总体要求和目标任务

总体要求是:以邓小平理论和“三个代表”重要思想为指导，深入贯彻落实科学发展观，坚定不移地贯彻“49字”工作方针，坚定不移地落实“123456”总体布局，坚定不移地坚持“高效生态”发展定位，准确把握主题主线，抢抓“两区”开发机遇，深入实施追赶超越战略，奋力建设生态滨州、美丽滨州、幸福滨州。

今后五年，要立足新起点，谋求新发展，实现新跨越。立足新起点，就是定位“黄蓝”两区，立足环渤海，放眼国内外，找准新坐标，谋划更科学的思路，制定更高远的目标。坚持:突破北部沿海不动摇；抢抓重大项目不动摇；产业融合升级不动摇；改善民计民生不动摇；创新体制机制不动摇。谋求新发展，就是按照市第八次党代会的部署要求，到2016年，实现地区生产总值、地方财政收入、城镇居民人均可支配收入、农民人均纯收入、固定资产投资、进出口总额“六个翻番”。重点实施“六项工程”。

(一)“沿海突破”工程

展望全球，世界发达国家无一不是以海港为核心，大力发展临港产业，带动整体经济发展。着眼滨州，我们差距在沿海，潜力在沿海，希望也在沿海。尤其是经过近几年举全市之力建设北海新区，沿海开发已蓄势待发。我们应该借鉴先进经验，做大做强沿海经济，努力实现北部崛起。

当前，经济发展的一个重要趋势是全球化，港口是交通的枢纽、是各种交通工具的转换中心，不仅能在周边大力发展加工业、物流业，更重要的是由于海运运量大、效率最高、成本最低，可以有效配置全球资源。随着滨州港2个3万吨级码头的开航运营，打通了我们走出大海、迈向世界的坦途，筑起了借海振兴、依海崛起的平台。要立足滨州、服务鲁西北、对接滨海新区，让滨州港早日成为全市经济社会发展的强大引擎。

未利用地是我们最大的优势和潜力，必须从战略和全局的高度加以把握，以黄河三角洲建设开发集团为平台，市县通力协作，加快推进确权、迁占、收储等工作，进一步做好核心区、主体区、联动区重点示范园区和示范基地建设，为北部乃至全市发展开辟新的空间，真正把这一最具潜力的优质资源，打造成支撑滨州跨越赶超的“黄金板块”。

进一步完善港口基础设施，扩大延伸港口腹地，优化顶层设计，明确功能定位，突出高效、集约要求，发展壮大油盐化工、装备制造、粮油加工、临港物流等临港产业，全力打造沿海经济隆起带。

　　(二)“产业提升”工程

树立“融合发展”理念，打破产业界限，探索转型升级新模式，推动三次产业相互融合，创造更多新的经济增长点。坚持“产业规划”全市统筹，全面构筑功能分区合理、资源集约利用、产业相对集中的空间布局。

突出高效生态农业，加快黄河三角洲（滨州）国家农业科技园区建设步伐，推动发展先进科技服务、现代物流信息、高端食品加工、现代农业高新技术研发，实现物联网技术应用示范、农业科技集成、产业链创业、农业高端服务四大功能。加快建设高效生态园区，带动发展绿色农产品生产、粮棉高产创建、荒碱地治理与高效种养、生态水产养殖等。加快发展设施农业、精准农业，增强综合生产能力。稳妥推进土地流转，积极发展规模经营，加快推动农业生产的标准化、产业化。

立足发展环境友好型工业，积极运用信息技术、高新技术改造提升传统产业，推动产业链条向两端延伸、高端攀升，加快建设家纺服装、油盐化工、装备制造、食品深加工“四大产业基地”。落实国家战略性新兴产业规划及省里的配套政策，鼓励企业技术创新、新产品研发、品牌创建，大力发展先进装备制造、新能源、新材料、新信息以及生物医药、节能环保等产业，力争重点领域的技术水平和产品档次大幅度提升。

着眼发展现代服务业，优化财税收入结构。大力发展电子商务、冷链物流、连锁配送、网上销售等现代流通业态，构建现代流通体系。积极发展金融组织，做大做强银行、保险、证券三大产业。以湿地、自然生态景观、高效生态农业旅游为主要内容，打造沿海生态旅游度假带、沿黄文化与休闲农业旅游带；突出园林化、生态化、特色化，打造黄河三角洲中心旅游城市、亲水园林生态旅游城市。

(三)“品牌拓展”工程

围绕黄河三角洲中心名城目标，统筹规划，搞好对接，搭建“一主三辅”临海傍河的城市主体框架。加快城市水系、景观、绿带、路网、管线建设，实施城中村改造，完善公共交通、市政公用和公共服务设施，推进“城市生态化、规划国际化、建设精品化、管理数字化”，提升“四环五海、生态滨州”建设水平。

构建布局合理、结构优化、安全畅通、四通八达的大路网体系，国家级公路运输主枢纽城市地位更加巩固。推进市县政府驻地一级路连接线和农村公路网络建设，打造滨州“四十分钟交通圈”。

　　围绕“粮丰林茂、北国江南”目标定位，持续开展“林水会战”。实施“五大生态示范工程”，构建以通道绿化为框架、镇村绿化为主体、生态林场为节点的现代绿化生态体系。抢抓国家加快水利改革的大好机遇，加快大型灌区续建配套与节水改造工程建设，构建引黄供水、防洪除涝、生态景观“三大体系”。抓好路域开发治理，完善农村交通网络，推进土地综合整治，加快农村住房建设及危房改造，逐步实现城乡规划、建设、管理、绿化、水务“一体化”，提升农村生态文明水平。

(四)“改革驱动”工程

突出发展县域经济，立足优势资源和产业基础，加快基础设施建设，提升园区承载能力，吸引符合产业政策的大项目落地，培育特色产业，加快建设经济强县。

实施产业园区带动战略，加快形成“以国家级园区为龙头、省级开发区为骨干、特色产业园区为支撑”的园区体系。坚持招商引资与招才引智相结合，加强与跨国集团、科研院所及国内外知名大学的合作，扩大招商引资规模，提高利用外资的水平和质量。深化国有企业改革，完善国有资产监管体系，提升国有资产监管质量和运营效益。加快财税制度改革，正确处理“保障运转、改善民生、支持发展”三者关系，完善公共财政体系。

(五)“生态支撑”工程

加大投入力度，加强自然保护区、生态湿地的保护与管理，完善沿海、沿黄生态防护屏障。建立资源节约集约利用与保护长效机制，合理开发水资源，集约利用土地资源，科学使用海域资源。

严格落实环境保护目标责任制，强化重点区域、重点流域、重点行业、重点企业污染治理，提高城镇垃圾、污水集中处理水平，有效控制农村面源污染，提高生态环境承载力。

强化节能减排，发展循环经济、绿色经济，以示范企业为带动，以清洁生产为手段，以经济园区为载体，推行集聚生产、集中治污、集约发展，努力打造全国循环经济发展示范区。

(六)“民生保障”工程

坚持教育优先发展，深入推进素质教育，积极发展学前教育，稳步发展高等教育，大力发展职业教育，努力提高高中教育质量。完成中小学校舍安全工程和标准化建设。财政教育支出占财政支出比重逐年增长，实现县域内义务教育均衡发展。

深化文化体制改革，探索建立国有文化资产管理中心，稳妥推进经营性文化单位转企改制，逐步实现政府由“办文化”向“管文化”的转变。加强公共设施建设，完善市县乡村服务体系，培育壮大文化产业，促进文化事业繁荣发展，积极建设“文化强市”。大力发展群众体育，提升竞技体育水平，全面增强人民体质。

深化医药卫生体制改革，初步建立覆盖城乡居民的基本医疗卫生制度，基本实现农村卫生社区化、城乡卫生一体化、医疗服务规范化、基本公共卫生服务均等化。切实做好食品药品安全工作。坚持计划生育基本国策，稳定低生育水平，降低出生人口性别比。

深入推进社会管理创新，健全完善立体化社会治安防控体系和网格化社会矛盾调处体系，积极构建和谐劳动关系，努力解决影响安全稳定的突出问题，促进社会公平正义。

健全完善社会保障体系，扩大就业覆盖面，大力发展社会保险、救助、福利事业，提高社会养老、基本医疗、最低生活保障、安居保障水平。

高度关注改善民生，自我加压，倾力而为，每年新增财力的80%要用于民生。滨州虽然地处“山东西部”，但在民生保障水平上，逐步按东部地区标准执行，努力让人民群众生活得更加殷实，更加幸福，更有尊严！

实现新跨越，就是坚定不移地走科学发展、内涵发展、可持续发展之路，着力构建“三大板块”。

南部跨越。南部(邹平、博兴、高新区)突出“高”和“新”，以邹平国家级经济技术开发区为龙头，以博兴经济开发区和高新区为主体，继续壮大经济总量，提高发展质量，全面推动传统产业改造提升和新兴产业快速崛起，打造国内新材料产业基地、装备制造业加工和研发基地、高端服务业聚集区。到2016年，南部地方财政收入实现150亿元;邹平、博兴综合实力分别达到全国前10位、全省前30位。

中部突围。中部(滨城、惠民、阳信、滨州经济开发区)突出“绿”和“特”，坚持生态优先，严格控制工业开发强度，推动滨州经济开发区争创国家级经济技术开发区，加快山东滨州工业园、惠民经济开发区和阳信经济开发区发展。大力实施农业科技创新和成果转化推广，统筹规划黄河两岸10公里以内区域，打造50处绿色和有机农产品示范基地，集中建设高效生态农业示范带。到2016年，滨城区综合实力进入全省前25位;惠民、阳信综合实力进入全省中游水平;滨州经济开发区综合实力进入全省省级开发区前5位。

北部崛起。北部(沾化、无棣、北海经济开发区)突出“大”和“强”，发挥临港优势，加快建成我市临港产业聚集区、山东省重要沿海经济增长带。立足沿海和未利用地优势，运用市场、税收等杠杆，探索生态补偿机制，通过承接产业转移和优势互补，实现生态与产业平衡发展，缩小南北差距。到2016年，无棣、沾化综合实力进入全省中上游水平，北部的地方财政收入达到70亿元。

各位代表，经过全市上下励精图治，顽强拼搏，滨州已经站到一个新的更高起点上。我们坚信，再经过五年的努力，“提前全面进入小康社会、再造一个新滨州”的美好愿景，一定能够早日实现！

三、2012年的重点工作

2012年是实施“十二五”规划承上启下的重要一年，是推进“两区”深度开发的关键之年，更是本届政府的开局之年。我们将牢牢把握稳中求进、好中求快的总基调，尊重科学规律，加快转调步伐，努力实现经济社会平稳较快发展。

预期目标是:地区生产总值增长12%，地方财政收入增长15%，固定资产投资增长22%，规模以上工业增加值增长15%，进出口总额增长12%，社会消费品零售总额增长17%，城镇居民人均可支配收入增长13%，农民人均纯收入增长13.5%，人口自然增长率控制在5.7‰以内，城镇登记失业率控制在3.6%以内。万元地区生产总值能耗及主要污染物排放，全部完成省里下达的目标任务。

　(一)全力推进“两区”开发建设

统筹搞好沿海产业规划，实现园区“全覆盖”，努力建设国家级循环经济示范区、环渤海物流中心和油盐化工、船舶制造、清洁能源、生物制药等产业聚集区。

整合北部港源，壮大滨州港务公司，加快港口附属及续建工程建设，上半年2个3万吨级码头开航运营。

德大铁路(滨州段)年内铺轨，寿邹铁路(滨州段)、黄大铁路(滨州段)、德大铁路滨州站、滨港铁路二期、滨海大道等工程开工建设。积极争取济南—滨州高速公路、济滨城际铁路、环渤海高等级公路早日开工。

细化分解未利用地开发任务，倒排工期，加快收储，完成起步区12.5万亩土地开发整理。启动徒骇河雨洪水资源综合利用工程。

完善招商选资政策，制定《产业招商实施办法》，精心组织20个境内外重要展会，积极引进世界500强、全国500强企业。制定出台“飞地”政策，建立跨区域重大项目落地机制。严把投资强度、环保系数、科技含量、企业规模等标准，对不符合要求的坚决拒之门外。

突出抓好40家进出口企业的调度指导，稳固纺织、服装等15种重点产品出口规模。精心组织银企合作促进会，优化存贷款结构。积极推进企业上市，加快引进股份制商业银行。深化预算管理改革，加快构建经营财政体系。

(二)全力推进产业转型升级

强化“园区带动”，加强与杨凌农业高新科技产业示范区、北京现代农业科技城的交流合作，加快黄河三角洲（滨州）国家农业科技园区建设步伐。加大71家高效生态农业示范园区培植力度，完成黄河两岸10公里区域统一规划，推进10个高效生态农业示范基地建设。实施“龙头拉动”，加快建设标准化、规模化、产业化生产基地，搞好4处农业部定点批发市场升级改造，做大做强4个国家级、53个省级龙头企业。坚持“科技推动”，实施种业科技振兴规划、农业良种及粮食丰产科技工程，加快农业物联网和农产品安全追溯体系建设。完成县区兽医体制改革，实现重大动植物疫病有效防控。

坚持“双轮驱动”，重点抓好400个投资过千万元的工业项目、60个“两化融合”项目、总投资480亿元的技改项目。加快建设黄河三角洲研发设计中心、省级技术中心、工业设计中心，提高自主创新能力。强化信用担保、人才培训、信息平台等服务体系建设，支持中小微企业发展。实施30项节能技改创新工程和10大循环经济示范项目，确保完成节能减排任务。

强化服务业“四大载体”建设，加快建设鲁北物流中心、黄河三角洲金融服务中心、环渤海南岸商贸信息服务中心。出台鼓励政策，加快企业二三产业剥离。突出抓好23个过亿元旅游业重点项目。

(三)全力推进城镇化进程

高起点编制总体性规划，完善城市基础设施和服务功能，加强地下空间规划和利用，开工垃圾焚烧发电、中水回用项目，加快道路、防汛、供暖、消防等公共基础设施建设，抓好城中村改造、老旧小区及市场周边环境整治，积极争创国家园林城市、全国文明城市。

强化金融、土地、人才支撑，扶持民营企业，发展实体经济。加大“突破惠民阳信”力度。在长山镇列为全省强镇扩权试点的基础上，扩大试点范围，提高城镇化水平，壮大县域经济。

深化“粮丰林茂、北国江南”品牌建设，扎实开展“林水会战”。新造林17万亩，实现24条干线道路绿色通道“全覆盖”，林木覆盖率增长1.2个百分点;完成水利工程466项，新增蓄水能力4802万方。

加大农村环境整治力度，推进生态文明村建设。加快实施10条骨干路域综合开发治理，积极推进合村并居，建设50处新型农村社区。统筹抓好农村电网改造、饮水安全、垃圾处理、沼气入户等工作。

(四)全力推进社会事业发展

鼓励多元化资金进入文化产业领域，提高市级文化产业发展专项资金增长幅度，加快市民文化中心、活动中心、公共卫生中心、健身中心以及滨州黄河三角洲文化产业园、西王玉米文化产业园、滨州海瓷艺术创意文化产业园、滨州云计算中心等基础设施建设。加快8家国有文艺院团改制步伐。积极培育文化市场，挖掘传承优秀传统文化。

落实积极的就业政策，加强职业培训和创业引领，促进高校毕业生、农村转移劳动力、城镇就业困难人员、退役军人等重点群体就业。继续增加退休人员基本养老金，完善农村和城镇居民基本养老保险制度，启动完善城镇职工基本医疗、工伤和失业保险市级统筹，努力提高“五保户”供养水平。

落实教育惠民政策，不断增加教育投入，财政教育支出占财政支出的比重达到20.1%以上。支持高等院校重点项目建设。推进医药卫生体制改革。落实计生家庭各项扶持政策。

牢固树立人才是第一资源的观念，落实《滨州市支持人才初始创业办法》，积极培养、吸引、用好人才，重点引进科技创新和产业领军人才。

全面落实支农惠农富农政策，推进扶贫开发，减轻农民负担，增加农民收入。建立维护农民工权益长效机制，解决农民工工资问题。关爱农村留守儿童健康成长。

创新社会管理，探索新时期改进群众工作的方式方法，强化安全生产和隐患排查治理，加大食品药品监管力度，预防和化解民间借贷风险，严厉打击各类违法犯罪活动，深入推进“平安滨州”建设。

发展慈善事业，做好希望工程救助、扶残助残工作，加快市残疾人康复中心建设。落实优抚政策，搞好“双拥共建”。

实施“收入倍增”计划，建立职工工资正常增长机制，稳步提高最低工资标准，着力提高农村居民、企业职工特别是低收入者和困难家庭收入。

在此基础上，集中办好16件关系群众切身利益的民生实事：

（1）加大财政投入，实现市城区所有中小学“平安校车”全覆盖，加快县区“平安校车”推进步伐。

（2）全力推进中小学校舍安全工程和标准化建设，投资2.8亿元，加固中小学校舍31万平方米、新建中小学校舍14万平方米。

（3）继续实施学前教育三年行动计划，投资2.6亿元，新建、改扩建幼儿园118处。

（4）投资8700万元，资助4.6万名家庭困难学生。

（5）建设保障性住房6920套，全面完成保障性住房建设任务。

（6）提高住房公积金抵押贷款和信用贷款额度，由原来的25万元、15万元，分别提高到30万元、20万元，还款期限由15年、10年，分别延长至20年、15年。

（7）开展供热计量改革，凡具备计量条件的居住建筑，全部取消按面积收费，实行按实际用热量计价收费。

（8）建立“环卫保洁全城覆盖，垃圾清运全城集中，垃圾处理全城统一”工作格局，全市60%的乡镇实现城乡环卫一体化管理。

（9）推进公交便民畅通工程，继续更新车辆，科学布局线路，新辟调整公交线路10条。

（10）开展各类就业、创业培训6万人，新增城镇就业3.5万人，农村劳动力转移就业5万人。

（11）加快市县养老服务中心建设，社会养老床位达到1.1万张以上，农村五保集中供养率稳定在80%以上。

（12）落实10大类41项基本公共卫生服务项目，居民电子健康档案建立率达到80%以上，新农合和城镇居民医保补助标准提高到每人每年240元。

（13）实施“万村千乡”市场工程，新建和改造农家店500家、配送中心2家。

（14）新改建农村公路1343.5公里，改造农村公路危桥30座。

（15）投资2.4亿元，完成无棣仝家河、沾化马新河等5条国家重点地区中小河流治理任务，完成惠民土马沙河、博兴支脉河等5项省规划重点河道治理工程。

（16）县级文化馆、图书馆全面达到国家二级馆以上标准，各乡镇（街道）全部建成符合国家规定标准的综合文化站，社区、行政村实现文化活动室、文化大院、农家书屋基本覆盖，企业职工书屋、文体活动中心等阵地建设提档升级。

各位代表，我们每年都要办好一批看得见、摸得着的民生实事，并持之以恒地坚持下去，让滨州人民的生活水平一年更比一年好！

(五)全力推进政府自身建设

打造“阳光政府”:大力推进政务公开，让行政权力始终在阳光下运行，依法、及时、准确、全面公开群众关心的政府信息。始终坚持公开是原则，不公开是例外，加强政府网站和市长公开电话工作，探索运用政府微博等新媒体，更好地回应社会关切的问题，进一步拓宽群众的知情权、参与权、监督权。

打造“责任政府”:公平、正义、诚信的缺失，必令社会失去方向，民众失去信心。每一位政府工作人员要时刻牢记：我们的权力是人民赋予的，权力只能用来为人民服务，受人民最严格的监督，对人民常怀敬畏之心，勇于负责，敢于担当，让人民群众愿意向政府提出意见、表达诉求，进一步提升政府公信力，努力建设人民信任的政府！

打造“效能政府”:提高依法行政水平和行政审批效率，大力实施“效能提升工程”。狠抓绩效管理，加大考核力度，严肃行政问责，营造争先创优的工作氛围。强化工作落实，不搞花架子，不搞形象工程，为滨州发展和人民幸福，办真事、办实事、办好事。

打造“廉洁政府”:任何缺乏监督和制约的权力都容易导致腐败，要规范公共资源交易，严格实行重大项目行政审批、施工操作、资金投入相互隔离，从源头上遏止“暗箱操作”。坚决打击以权谋私、执法不公、部门牟利等行为，决不允许不廉洁行为影响和干扰发展大局，以反腐倡廉的实际成效树立形象、取信于民。

各位代表，面对新征程新目标、新任务新要求，我们责无旁贷，义不容辞!我们坚信，只要我们心里装着百姓、胸中怀有大局，就一定会把责任看得重于泰山;只要我们倾情工作，殚精竭虑，滨州事业就一定会欣欣向荣、蒸蒸日上!

各位代表，让我们在中共滨州市委的坚强领导下，紧密团结在以胡锦涛同志为总书记的党中央周围，进一步振奋精神，锐意进取，奋力夺取全面建设小康社会的新胜利，以优异成绩迎接党的十八大和省第十次党代会胜利召开！
